# Hemophagocytic Lymphohistiocytosis Secondary to Adult-Onset Still’s Disease

**DOI:** 10.7759/cureus.18037

**Published:** 2021-09-17

**Authors:** Bhupen Barman, Md Jamil, Pranjal Kalita, Biswajit Dey

**Affiliations:** 1 Internal Medicine, North Eastern Indira Gandhi Regional Institute of Health and Medical Sciences (NEIGRIHMS), Shillong, IND; 2 Pathology, North Eastern Indira Gandhi Regional Institute of Health and Medical Sciences (NEIGRIHMS), Shillong, IND

**Keywords:** cell-mediated immunity, secondary hemophagocytic lymphohistiocytosis, adult-onset still’s disease, arthritis, immunity

## Abstract

Hemophagocytic lymphohistiocytosis (HLH) is a rare hematological condition resulting from dysregulation of the immune system. This unusual clinical syndrome is characterized by fever, cytopenia, liver dysfunction, increased ferritin level, and evidence of hemophagocytosis in the bone marrow. We report a case of a 21-year-old female who presented with recurrent high-grade fever, transient rash, and polyarthritis who was subsequently diagnosed with adult-onset Still’s disease (AOSD) with secondary HLH. The patient improved with aggressive management. Our case highlights HLH as a life-threatening and underdiagnosed complication of AOSD.

## Introduction

Hemophagocytic lymphohistiocytosis (HLH) is a potentially fatal hyperinflammatory syndrome that is characterized by histiocytic proliferation and hemophagocytosis [1]. This condition has two forms: primary HLH (familial) and secondary HLH (reactive). Secondary HLH which is also called macrophage activation syndrome (MAS) is usually acquired following infection, malignancy, and autoimmune diseases [2]. Presenting features include fever, hepatosplenomegaly, and cytopenias. A high index of clinical suspicion is required for diagnosis including a search for the precipitating factor. The criteria for diagnosis of HLH published in 2004 require the presence of five of eight of the following: fever; splenomegaly; cytopenia (affecting at least two of three lineages in the peripheral blood); fasting triglyceride levels ≥3 mmol/L and/or fibrinogen level ≤1.5 g/L; serum ferritin level ≥500 ng/ml; soluble CD25 level ≥2400 U/ml; decreased or absent natural killer (NK) cell activity (according to local laboratory reference); or hemophagocytosis in bone marrow, spleen, or lymph nodes [3]. The H-score of 2014 devised by Fardet et al. was a modification of the previous system and gave simplified numerical values to the various clinical, laboratory, and histological parameters which is comparatively easier and helps in prompt diagnosis of secondary HLH patients [4]. Here, we report a patient with secondary HLH complicating adult-onset Still’s disease (AOSD).

## Case presentation

A 21-year-old woman presented with a history of high-grade fever with chills and rigor on and off since the last one year and multiple small and large joint pain since the last 8 months which was initially limited to both knee joints, but subsequently progressed to involve both ankles, wrists, shoulder and multiple small joints of hands in a symmetric manner. The joint pain was associated with early morning stiffness lasting for more than one-hour duration which used to get relieved on physical activity. There was also a history of transient maculopapular rash mostly in limbs with spontaneous disappearance. She also complained of recurrent episodes of sore throat. There was no history of oral ulceration, alopecia, hemoptysis, leg swelling, early morning puffiness, and decreased urine output. The patient also did not give any history of pain over the back of the neck or any radiating pain. On examination, she was febrile with a temperature of 38.5°C (101.3°F), pulse rate of 120 beats/minute, and blood pressure of 110/70 mmHg. There was severe pallor with mild hepatosplenomegaly and bilateral cervical lymphadenopathy. There was evidence of active arthritis in wrist joints, multiple metacarpophalangeal (MCP) joints, and proximal interphalangeal (PIP) joints of both hands, both knee and ankle joints with mild restriction of mobility.

Laboratory parameters on admission showed a hemoglobin level of 5.7 g/dl, white blood cell count 12,600/µl with 83% neutrophils, 14% lymphocytes, and 3% monocytes, a platelet count of 78,000/mm3, and erythrocyte sedimentation rate (ESR) of 120 mm/1st hour. Peripheral blood picture showed microcytic hypochromic anemia with neutrophilic leukocytosis. Serum chemistry including kidney function tests and liver function tests were within normal range. Her fasting lipid profile showed total cholesterol of 202 mg/dl (normal range [NR]: 0-200 mg/dl), triglycerides of 441 mg/dl (NR:30-200 mg/dl), high-density lipoprotein (HDL) of 30 mg/dl (NR:0-60 mg/dl), and low-density lipoprotein (LDL) of 107 mg/dl (NR: 0-130 mg/dl). Her serum ferritin was >15,000 ng/ml and her serum iron was 19.8 µg/dl (NR: 50-150 µg/dl). Her serum fibrinogen level was 369 mg/dl (NR: 233-496 mg/dl) (Table 1).

**Table 1:** Laboratory data of the patient

Variable	On Arrival at This Hospital (Day 1)	On discharge (Day 7)	Follow-up after one month	Reference Range for Adults
Hematocrit (%)	17.1	22.8	32.4	35.4-44.4
Hemoglobin (g/dl)	5.7	7.6	10.8	12.0-15.8
White-cell count (per mm^3^)	12,600	9800	7800	4000-11,000
Differential count (%)				
Neutrophils	83	75	74	40-70
Lymphocytes	14	20	22	20-50
Monocytes	03	03	04	4-8
Eosinophil	00	02	00	0-6
Basophils	00	00	00	0-2
Platelet count (per mm^3^)	78 х 10^3^	110 х 10^3^	225 х 10^3^	165-415 х 10^3^
Erythrocyte sedimentation rate (mm/hr)	120	78	42	0-20
Blood urea (mg/dl)	36	22	16	7-20
Serum creatinine (mg/dl)	1.2	0.8	0.8	0.5-0.9
Total Cholesterol (mg/dl)	202	198	176	0-200
Triglycerides (mg/dl)	441	202	156	30-200
Low density lipoprotein (mg/dl)	107	104	98	0-130
Serum glutamic-oxaloacetic transaminase (IU/L)	55	35	30	4-45
High density lipoprotein (mg/dl)	30	36	43	0-60 mg
Serum Ferritin (ng/ml)	15000	4500	205	12-150 ng/ml
Serum Fibrinogen (mg/dl)	369			233-496
C Reactive Protein (mg/dl)	161	56	9.8	< 10

**Figure 1:**
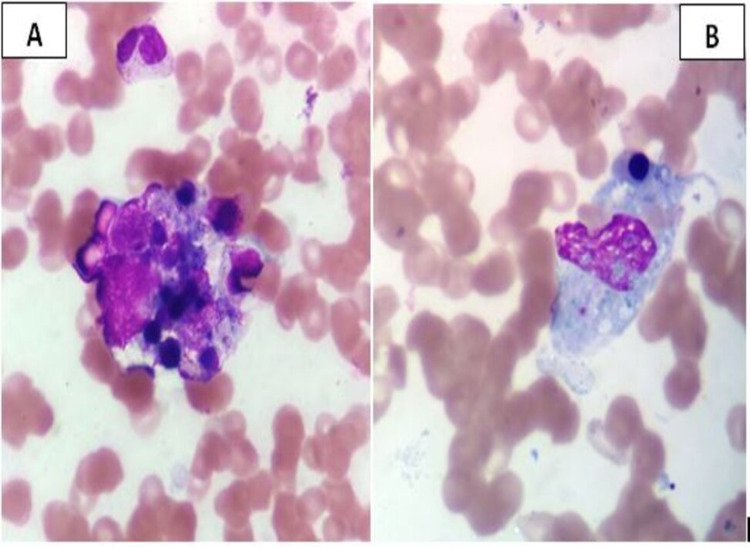
A) Bone marrow aspirate showing histiocyte phagocyting myeloid and erythroid precursor cells; B) histiocyte showing erythrophagocytosis (Leishman stain, 1000x)

Immunological studies showed a high rheumatoid (RA) factor titer of 22.2 IU/ml (NR: <15 IU/ml), the normal value of anti-cyclic citrullinated peptide (CCP), and a very high C-reactive protein (CRP) level of 161.0 mg/L (NR: <10 mg/L). Her anti-neutrophilic antibody (ANA) was negative by immunofluorescence method and serology for hepatitis A, B, C, cytomegalovirus (CMV), Epstein-Barr virus (EBV), herpes simplex virus, Toxoplasma, Salmonella, Leptospira, Brucella, Leishmania, malaria, dengue, and the human immunodeficiency virus (HIV) were negative. The patient tested negative for severe acute respiratory syndrome coronavirus 2 (SARS-CoV-2) by reverse transcription-polymerase chain reaction (RT-PCR). Her bone marrow aspiration showed evidence of hemophagocytosis (Figure 1).

Her ultrasonography of the abdomen showed grade I fatty liver with mild hepatomegaly and splenomegaly. There were no abnormal findings in her chest X-Ray and computed tomography of the chest and her upper gastrointestinal endoscopy were suggestive of antral gastritis. All routine cultures of blood and urine were normal. The H-score estimated as per the H-score of 2014 was 263 points with a probability of secondary HLH to be >99% (Table 2).

**Table 2:** H-score-2014 of the patient HLH: hemophagocytic lymphohistiocytosis

Parameters	Number of points (criteria for scoring)	Scoring of the patient
1. Known underlying immunosuppression	0 (no) or 18 (yes)	0
2. Temperature (°C)	0 (<38.4), 33 (38.4–39.4), or 49 (>39.4)	33
3. Organomegaly	0 (no), 23 (hepatomegaly or splenomegaly), or 38 (hepatomegaly and splenomegaly)	38
4. Number of cytopenias	0 (1 lineage), 24 (2 lineages), or 34 (3 lineages)	24
5. Ferritin (ng/ml)	0 (<2,000), 35 (2,000–6,000), or 50 (>6,000)	50
6. Triglyceride (mmoles/liter)	0 (<1.5), 44 (1.5–4), or 64 (>4)	64
7. Fibrinogen (gm/liter)	0 (>2.5) or 30 (≤2.5)	00
8. Serum glutamic oxaloacetic transaminase (IU/liter)	0 (<30) or 19 (≥30)	19
9. Hemophagocytosis features on bone marrow aspirate	0 (no) or 35 (yes)	35
Total H-score		263
Probability of secondary HLH		>99%

The patient was started on intravenous methylprednisolone 1000 mg/day for three days followed by oral prednisolone 40 mg/day and tapered off gradually to 10 mg/day. Her symptoms recovered rapidly with the patient becoming afebrile by the second day of intravenous steroids. She received two units of packed cell transfusion during her hospital stay. The patient was discharged with the above treatment regimen and was doing well on follow-up after one month of discharge with a normal complete blood count, serum ferritin of 205 ng/ml, ESR of 42 mm/1st hour, and CRP of 9.8 mg/L.

## Discussion

Still’s disease is a rare clinical syndrome characterized by a triad of high-grade fever, polyarthritis, and evanescent skin rash. AOSD is a clinical entity that was first described by Bywaters in 1971 and has varied clinical expression and unpredictable progression [5]. HLH is a rare life-threatening complication of Still’s disease. The present case met the criteria for AOSD and HLH. On admission, she had recurrent fever with a temperature of >38°C on several occasions, moderate hepatosplenomegaly, bicytopenia, hypertriglyceridemia, and hyperferritinemia, leading to a clinical diagnosis of HLH. Bone marrow aspirate of the patient showed hemophagocytosis. As per the H-score devised by Fardet et al., the optimal cut-off for a diagnosis of HLH was considered to be 169 with 93% sensitivity and 86% specificity [4,6]. In the present case, the H-score was 263 points, which predicts a more than 99% probability of hemophagocytic syndrome.

Rheumatoid arthritis and systemic lupus erythematosus are two important differential diagnoses in this case but the presence of recurrent fever, rash, sore throat, and lymphadenopathy pointed towards a clinical diagnosis of AOSD. The lack of a significant family history indicating immunodeficiency suggests that the HLH, in our patient, belonged to a secondary or reactive HLH. Secondary HLH is mostly associated with hematological malignancies (in particular T-cell lymphoma), different infectious diseases (viruses like EBV, CMV, dengue virus, bacteria, leishmaniasis, fungi), and drugs (methotrexate, sulfasalazine, and anti-TNF-ɑ like etanercept, infliximab), as well as other autoimmune diseases like systemic lupus erythematosus, systemic-onset juvenile idiopathic arthritis, and rheumatoid arthritis [7-9]. All these factors may also overlap with AOSD as it is itself postulated to be autoimmune.

Association of HLH and AOSD has been described in two case series with an estimated prevalence of HLH to occur in 7%-15% of patients with AOSD [10,11]. The characteristics of the patients in both the case series include fever, lymphadenopathy, rash, hepatosplenomegaly, arthralgia, and sore throat with bi/pancytopenia and high CRP and an increase in blood levels of bilirubin, transaminases, and LDH. Most striking and specific is the extremely high serum ferritin value that was found here. Although the cause of the HLH is unknown, dysregulation of macrophage-lymphocyte interactions with subsequent increases in the levels of both T-cell-derived and macrophage-derived cytokines, particularly TNF-ɑ, interleukin (IL)-1, IL-6, interferon-gamma (IFN-ƴ), soluble IL-2 receptor (sIL-2R), and soluble TNF receptors (sTNFRs) could be involved in this syndrome [12]. Such dysregulation leads to an intense systemic inflammatory reaction.

A very high serum concentration of ferritin appeared to be the key to diagnosis in patients with AOSD [13]. The fact that such a finding actually only occurs with HLH and AOSD suggests a relationship between the two diseases. It could be hypothesized that AOSD is a mild variant of the HLH. This hypothesis is supported by Min et al. in which 17% of the total 12 AOSD patients without the active disease had evidence of hemophagocytosis in the bone marrow [14].

## Conclusions

HLH occurring in the course of rheumatic diseases is an important and often underdiagnosed clinical entity that requires a high index of suspicion for diagnosis. Patients with high-grade continuous fever associated with hepatosplenomegaly and lymphadenopathy should be evaluated for HLH. This case suggests that AOSD may precipitate HLH and treatment of the primary disorder, as in this case scenario, may be lifesaving.
